# Study on health hazards through medicines purchased on the Internet: a cross-sectional investigation of the quality of anti-obesity medicines containing crude drugs as active ingredients

**DOI:** 10.1186/s12906-015-0955-2

**Published:** 2015-12-04

**Authors:** Naoko Yoshida, Midori Numano, Yoko Nagasaka, Kaori Ueda, Hirohito Tsuboi, Tsuyoshi Tanimoto, Kazuko Kimura

**Affiliations:** Drug Management and Policy, Faculty of Pharmacy, Institute of Medical, Pharmaceutical and Health Sciences, Kanazawa University, Kakuma-machi, Kanazawa, Ishikawa 920-1192 Japan; Faculty of Pharmaceutical Sciences, Doshisha Women’s University, Kodo, Kyotanabe, Kyoto 610-0395 Japan

**Keywords:** Suspended medicines, Diet aid products, Anti-obesity medicines, Sibutramine, Personal import, Internet

## Abstract

**Background:**

Weight-loss medicines, including crude drugs and herbal supplements disguised as diet-aid products, are readily obtainable and distributed widely, especially in Southeast Asia. Even if such products are unapproved or prescription-only medicines, consumers can purchase them through an agency or directly on the Internet. We evaluated the quality and safety of herbal products purchased on the Internet to reveal their influence on public health.

**Methods:**

Diet-aid products containing Bukuryo (*Poria sclerotium*), Bakumondo (*Ophiopogonis* tuber), or Daio (rhubarb rhizome) were purchased through websites that did not provide physical addresses or which advertised misleading medicines (e.g., unapproved Cialis 100 mg tablets, Viagra 100 mg tablets) on websites. We carefully noted details in the descriptions on package inserts or accompanying product characteristics and analyzed the ingredients using qualitative and quantitative methods, namely high-performance liquid chromatography equipped with a photodiode array detector. We requested the respective manufacturers to authenticate their products through a structured questionnaire.

**Results:**

We purchased 15 items from 15 Internet sites and imported all 15 items to Japan. One item stated to contain rhubarb rhizome was identified as a prescription medicine; the others were dietary supplements and not medicines. Even though we did not analyze the constituents of all crude drugs, we found some active ingredients in the items. Sibutramine was detected in items confirmed to be supplements, including those containing *Poria sclerotium* and *Ophiopogonis* tuber. Each capsule contained ≈ 12 mg of sibutramine, which is the daily dose for anti-obesity medicines. Sibutramine is not approved for use in Japan and its sale has been suspended in Europe and the USA owing to serious adverse effects on the circulatory system.

**Conclusions:**

Our findings indicate that dietary supplements containing injurious ingredients are distributed to Japanese consumers and potentially to a broader international audience, and that purchasing them through unreliable websites bears potential health risks. To avoid potential adverse events, there should be adequate alerts about the risks of taking products without appropriate indications.

## Background

The increasing international trade in pharmaceuticals *via* sales on the Internet has facilitated the entry of counterfeit products into the supply chain. Counterfeit products include products with ingredients different from those labeled or advertised, without active ingredients, with insufficient or excessive active ingredients, and with misleading packaging [1]. Since 2011, the World Health Organization (WHO) has suggested that counterfeit medicines be termed “spurious/falsely labelled/falsified/counterfeit” (SFFC) medicines [2]. In parallel, Attran and colleagues recommended reserving the term “falsified” for crimes against public health [3]. They defined “falsified medicines” as medicines that violate the quality specifications of the regulator—but what truly defines and distinguishes them is criminal intent [3]. Use of such medicines can result in treatment failure or even death. The WHO has warned that medicines purchased over the Internet from sites that conceal their physical addresses are falsified in >50 % of cases [1]. An individual can import restricted quantities of medication for personal use without undergoing special procedures in Japan [4]. Accordingly, pharmaceutical regulatory authorities in many countries and regions such as the Ministry of Health, Labour and Welfare in Japan [5], the US Food and Drug Administration [6, 7], European Medicines Agency [8], Medicines and Healthcare Products Regulatory Agency in the United Kingdom [9, 10], Health Canada [11], and the Therapeutic Goods Administration in Australia [12], promote awareness regarding medicines that should be avoided and not purchased over the Internet.

Obtaining accurate information on the extent of falsified medicines is difficult because the falsified medicines detected are a small proportion of the total number of falsified medicines that are available. Because of this situation, sharing information and promoting awareness in regard to falsified medicines may help to combat the health hazards associated with the use of falsified medicines. Preventing health hazards is a societal requirement. It is necessary to avert the serious adverse effects of imported medicines by undertaking countermeasures supported by field-based and scientific evidence. Our research team has highlighted the public-health implications of privately importing medicines such as levonorgestrel and piracetam (which are prescription medicines in Japan) [13, 14].

Among privately imported medicines in Japan, anti-obesity medicines are the most popular, followed by medicines for erectile dysfunction and hair growth [15]. Reductil® contains sibutramine, and is one of the most popular anti-obesity agents found on websites, but it is not approved in Japan. In one Reductil-containing product imported *via* the Internet, the maximum dose of sibutramine described in the explanatory note in Japanese was nearly three-times the dose described in the true package insert, but this item was confirmed as “authentic” by the manufacturer [14]. In 2012, it was reported that a product labeled Xenical® (120 mg orlistat), a synthetic anti-obesity medicine imported *via* the Internet, had no active ingredient except starch. Furthermore, it was a falsified medicine according to the response from the manufacturer to our requests for authentication [16].

In the present study, we investigated the quality of samples of diet-aid products and weight-loss medicines purchased on the Internet using chemical analyses.

## Methods

### Sample collection

Using Japanese keywords for “personal import agent”, “slimming” and “obesity” (terms used in a list of “plant-origin materials used mainly as pharmaceutical medicine” created by the Ministry of Health, Labour and Welfare in Japan [17]), we searched for diet-aid products that were described on Internet sites as containing Bukuryo (*Poria*, *Poria sclerotium*), Bakumondo (*Ophiopogonis* tuber), or Daio (rhubarb rhizome) on the Japanese Google search engine (www.google.co.jp). Among the websites advertising such products, we selected those sellers that did not disclose their physical addresses and those that advertised the sale of falsified medicines (e.g., with unapproved dosages) in August 2009 to purchase the items mentioned above [16]. *Poria*, *Ophiopogonis* tuber and rhubarb have been approved for reimbursement under Japanese national health insurance as prescription and over-the-counter medicines. These crude drugs are used widely as diuretics or purging agents in Japan.

### Observation

We carefully noted the descriptions (ingredients, quantity, dosage form, package presentation, usage instructions, safety information, manufacture date, expiration date, batch number, name and address of the manufacturer) on the website and package insert or the summary of product characteristics of the items purchased.

### Authenticity investigation

The methodology of the authenticity investigation and registration verification was adopted from that provided by the WHO [16, 18]. We requested the respective manufacturers to authenticate their products through a structured questionnaire (including verification of the descriptions on the package, equivalence in appearance, and licensed status for manufacturing and marketing) sent by post together with a part or photograph of the items.

### Materials

We purchased benfluorex, orlistat, and aristolochic acid (mixture of I and II) from Sigma–Aldrich Co. LLC (Saint Louis, MO, USA). Lovastatin was purchased from Toronto Research Chemicals (Toronto, ON, Canada). We bought rimonabant from Cayman Chemical Company (Ann Arbor, MI, USA), and sibutramine hydrochloride from Tocris Bioscience (Minneapolis, MN, USA). We purchased fenfluramine and N-nitroso-fenfluramine from Wako Pure Chemical Industries, Ltd. (Osaka, Japan). These compounds were the reference standards of the compounds that were not listed on the package or insert. To verify that rhubarb rhizome was present, sennoside A (one of the anthraquinone glycosides in rhubarb and a compound used for identification of rhubarb rhizome according to the *Japanese Pharmacopoeia 16*) and authentic rhubarb rhizome were purchased from Nacalai Tesque, Inc. (Kyoto, Japan) and Tochimoto Tenkaido Co., Ltd. (Osaka, Japan), respectively. The reference standards of the other anthraquinone glycosides, aloe-emodin and emodin were purchased, respectively, from Toronto Research Chemicals and Tokyo Chemical Industry Co., Ltd. (Tokyo, Japan). Rhein and chrysophanol were bought from Extrasynthese S.A. (Genay, France). High-performance liquid chromatography (HPLC)-grade reagents of acetonitrile, 2-propanol, methanol, and trifluoroacetic acid were purchased from Wako Pure Chemical Industries, Ltd. All other chemicals were available commercially and of analytic grade. A membrane filter (Millex®-LG; pore size, 0.20 μm; Merck Millipore, Billerica, MA, USA) was used for filtration treatment for HPLC analyses. Thin-layer chromatography (TLC; silica gel 60 F254; 20 × 20 cm, Merck, Billerica, MA, USA) was employed for analyses.

### Sample preparation

We categorized purchased materials into three types of dosage form: soft capsules, hard capsules, and sugarcoated tablets. For soft capsule samples, we cut the capsules open, weighed the contents, transferred them to a 100-mL volumetric flask, and added an appropriate quantity of mobile phase. This solution was mixed by shaking for 90 s, passed through a membrane filter and analyzed. For hard capsule samples, we weighed the content in the capsule, which was sonicated with ≈ 80 mL of methanol for 30 min and diluted to 100 mL in a volumetric flask. The diluent was passed through a membrane filter and analyzed. For the sugarcoated tablet sample, we removed the coating with water. Thereafter, the whole of the uncoated tablet was sonicated with ≈ 80 mL of methanol for 30 min and diluted to 100 mL in a volumetric flask. The diluent was passed through a membrane filter and analyzed.

We dissolved each of the reference standards of sennoside A, aloe-emodin, chrysophanol, emodin, and rhein (1 mg) in 10 mL of ethanol. We passed each solution through a membrane filter and analyzed them.

To extract rhubarb rhizome, we pulverized adequate amounts of rhubarb rhizome. To 1 g of pulverized rhubarb rhizome, we added 10 mL of ethanol and then sonicated it for 10 min (2510 J-MF; Branson Ultrasonic Corporation, Danbury, CT, USA), and centrifuged it for 5 min (800 × *g*). The supernatant was passed through a membrane filter and analyzed using TLC.

To 200 mg of pulverized rhubarb rhizome, we added 4 mL of ethanol, sonicated it for 10 min, and centrifuged it for 5 min (800 × *g*). A diluent of 1 mL of supernatant with 3 mL of ethanol was filtered and injected into the HPLC system.

### HPLC analyses

The three HPLC methods described below were employed to detected benfluorex, lovastatin, rimonabant, sibutramine hydrochloride, orlistat, aloe-emodin, chrysophanol, emodin, rhein, aristolochic acid, fenfluramine, and N-nitroso-fenfluramine. To identify the compounds contained in purchased items, we confirmed that a single peak appeared upon simultaneous injection with the reference standard of the substance.

### System suitability

HPLC was undertaken according to the method described in the *Japanese Pharmacopoeia 16* [19]. Each product was analyzed before the expiry date detailed on the label.

### Method 1

Benfluorex, rimonabant, sibutramine hydrochloride and orlistat are well known synthetic anti-obesity compounds. Lovastatin is a cholesterol-lowering statin and has often been mistaken for having anti-obesity effects. We carried out quantitative determination of benfluorex, lovastatin, rimonabant, sibutramine hydrochloride, and orlistat with the calibration curves constructed for a HPLC system equipped with a photodiode array (PDA) detector (LC-20 AD; Shimadzu, Kyoto, Japan) according to the method for chemical analyses described in our previous study [16]. The analytical column was a Mightysil RP-18GP (4.6 × 150 mm, 5 μm; Kanto Chemical Co., Inc., Tokyo, Japan). The column temperature was maintained at 45 °C using a column oven (CTO-20A; Shimadzu). The mobile phase was a mixture of methanol and 0.02 mol/L phosphate buffer, pH 7.0, (17:3, *v/v*). The flow rate was 1.2 mL/min. The detection wavelength was 225 nm and we recorded ultraviolet (UV) spectra at 200–400 nm (SPD-M20A; Shimadzu). The injection volume was 20 μL. Under these conditions, benfluorex, lovastatin, rimonabant, sibutramine hydrochloride, and orlistat were eluted in that order and separated completely in the chromatogram (data not shown). The standard ingredient was identified by its retention time and absorption spectrum. To prepare solutions of standards, we dissolved benfluorex, lovastatin, rimonabant, sibutramine hydrochloride, or orlistat with methanol and diluted them stepwise to the appropriate concentration.

### Method 2

We detected aloe-emodin, chrysophanol, emodin, and rhein (the active ingredients of rhubarb rhizome) using the HPLC-PDA system. The analytical column was as described for Method 1. We maintained the column temperature at 45 °C using a column oven (CTO-10Avp; Shimadzu). For the mobile phase, we prepared a mixture of methanol, acetonitrile, and 0.1 % formic acid (8:1:1, *v/v/v*). The flow rate was 1.0 mL/min. We set the detection wavelength at 254 nm and recorded UV spectra at 200–400 nm (SPD-M10Avp; Shimadzu). The injection volume was 10 μL.

### Method 3

Aristolochic acid is a harmful compound found in Chinese herbal supplements that promote anti-obesity and which are imported *via* the Internet [20, 21]. Fenfluramine and N-nitroso-fenfluramine are also harmful compounds which have anti-obesity effects similar to those of sibutramine [22]. Chromatograms were recorded for the standard compounds of aristolochic acid, fenfluramine, and N-nitroso-fenfluramine using the HPLC-PDA system according to methods described in the *Japanese Pharmacopoeia 16* [19]. The analytical column was a Cosmosil C18-AR-II (4.6 × 150 mm, 5 μm; Nacalai Tesque, Inc.). We maintained the column temperature at 40 °C using a column oven (CTO-20 AC; Shimadzu). The flow rate was 1.0 mL/min. We recorded UV spectra at 200–400 nm (SPD-M10Avp; Shimadzu). The injection volume was 10 μL.

To detect aristolochic acid, a mobile phase (methanol and 0.02 M phosphate buffer at pH 7.0 (1:1, *v/v*)) was prepared. We set the detection wavelength at 310 nm. To detect fenfluramine and N-nitroso-fenfluramine, we prepared a mobile phase (methanol and water (4:6, *v/v*)) to which we added, respectively, 1 mL of trifluoroacetic acid and a mixture of methanol and 0.2 % trifluoroacetic acid solution (3:2, *v/v*). We set the detection wavelength at 210 nm.

### TLC analyses

We detected the ingredients in the sugarcoated tablet supposedly containing rhubarb rhizome and the extract of authentic rhubarb rhizome with reference to the monograph on rhubarb rhizome in the *Japanese Pharmacopoeia 16* [19] and compared them. We developed a plate with a mixture of 1-propanol, ethyl acetate, water, and acetic acid (40:40:30:1, *v/v/v/v*) to ≈ 40 mm. We observed spots under UV light at 254 nm (SLUV-6; 254/365 nm; As One, Osaka, Japan).

## Results

We purchased 15 items 11 products from six manufacturers sold on 15 Internet sites. Among the 15 websites, five lacked a description of their physical addresses, and four, one, and five sites advertised falsified Cialis, Levitra, and Viagra, respectively. These falsified products had unapproved content or dosage forms.

Of the 15 items, 11 items from eight products contained *Poria*, three items from one product contained *Ophiopogonis* tuber, and one item contained rhubarb rhizome. One item containing rhubarb rhizome was confirmed to be authentic and was a prescription medicine according to the reply from the manufacturer; the others were dietary supplements and not medicines. We noted the crude-drug components of each item listed in the descriptions on the outer packaging or package inserts (Table 1). For one item, no information about such components was provided.

**Table 1:** Item information

Item number	Product code	Product name displayed on package	Name of holder of marketing authorization	Country of holder of marketing authorization	Approval status	Ingredients displayed on package (scientific or common name)	Description of sibutramine containing	
							Package or insert	Website
1	A	韓国痩身1号(美体型)	西安華威保健有限公司	China	Supplement	山楂 (*Crataegi* fructus), 茯苓 (*Poria sclerotium*), 菜菔子 (*Raphani* semen)	No	No
2	B	終極痩身	西安華威保健有限公司	China	Supplement	蘭姫花, 櫻花素, 錦帯花, 珍珠粉 (pearl powder), 蘆薈 (*aloe*), 核桃油 (walnut oil)	No	No
3	C	韓国痩身1号(美腿型)	西安華威保健有限公司	China	Supplement	茯苓 (*Poria sclerotium*), 菜菔子 (*Raphani* semen), 苦宁素	No	No
4	D	韓国痩身1号(収腹提臀型)	西安華威保健有限公司	China	Supplement	山楂 (*Crataegi* fructus Fructus), 香茶素, 决明子 (*Cassiae* semen), 荷叶素	No	No
5	B	終極痩身	西安華威保健有限公司	China	Supplement	蘭姫花, 櫻花素, 錦帯花, 珍珠粉 (pearl powder), 蘆薈 (aloe), 核桃油 (walnut oil)	No	No
6	E	韓国痩身1号(新1代)(美体型)	西安華威保健有限公司	China	Supplement	山楂 (*Crataegi* fructus Fructus), 茯苓 (*Poria Sclerotium*), 菜菔子 (*Raphani* semen)	No	No
7	F	韓国痩身1号(新1代)(収腹提臀型)	西安華威保健有限公司	China	Supplement	山楂 (*Crataegi* fructus Fructus), 香茶素, 决明子 (*Cassiae* semen), 荷叶素	No	No
8	G	RELACORE	America Leptin Phrmaceuticals Limited	USA	Supplement	Not listed	No	No
9	H	代代花胶嚢	Kuming Dali Industry & Trade Co., Ltd.	China	Supplement	代代花 (*Citrus aurantii* flos)	No	Yes
10	I	妙姿	百年医葯集団有限公司	China	Supplement	山楂 (*Crataegi* fructus Fructus), 蘆薈 (Aloe), 枳実 (*Aurantii Fructus* immaturus), 桃仁 (*Persicae* semen)	No	No
11	J	韓国緑素抗脂胶嚢	珠海尚高乐实业发展有限公司	China	Supplement	奇昇果, 茶叶, 决明子 (*Cassiae* semen), 荷叶素, 山楂 (*Crataegi* fructus Fructus), 麦冬(*Ophiopogonis* tuber), 檳榔 (*Areca* semen), 青皮 (*Citrus unshiu* pericarpium immaturus)	No	No
12	J	韓国緑素抗脂胶嚢	珠海尚高乐实业发展有限公司	China	Supplement	奇昇果, 茶叶, 决明子 (*Cassiae* semen), 荷叶素, 山楂 (*Crataegi Fructus* Fructus), 麦冬(*Ophiopogonis* tuber), 檳榔 (*Arecae* semen), 青皮 (*Citrus unshiu* pericarpium immaturus)	No	No
13	J	韓国緑素抗脂胶嚢	珠海尚高乐实业发展有限公司	China	Supplement	奇昇果, 茶叶, 决明子 (*Cassiae* Cassiae semen), 荷叶素, 山楂 (*Crataegi Fructus*), 麦冬(*Ophiopogonis* tuber), 檳榔 (*Arecae* semen), 青皮 (*Citrus unshiu* pericarpium immaturus)	No	No
14	K	軽身減肥片	陕西君寿堂制药有限公司	China	Prescription medicine	大黄 (rhubarb rhizome), 防已 (*Sinomeni caulis et* rhizoma), 丹参 (*Salviae Miltiorrhizae* radix), 茵陜 (*Artemisiae Capillaris* herba), 沢泻 (*Alismatis* rhizoma), 山楂 (*Crataegi* fructus Fructus), 水牛角, 淫羊藿 (*Epimedii* herba), 黄芪 (*Astragali* radix), 白朮 (*Atractylodis* rhizoma), 川芎 (*Cnidii* rhizoma)	No	No
15	C	韓国痩身1号(美腿型)	西安華威保健有限公司	China	Supplement	茯苓 (*Poria sclerotium*), 菜菔子 (*Raphani* semen), 苦宁素	No	No

Items purchased from the 15 websites were sent by nine dispatchers, including one whose shipping label was indecipherable. All dispatchers were located in China. Of the six manufacturers, one was located in the USA, and the others were in China.

In the authenticity investigation, only one of the six manufacturers replied: the item containing rhubarb rhizome manufactured by the respondent was confirmed to be a genuine product according to the manufacturer’s reply. With three manufacturers, the questionnaires were returned because the address was incomplete, or the website provided on the outer packaging could not be accessed. Two e-mail addresses of these three manufacturers were not deliverable.

**Fig. 1:**
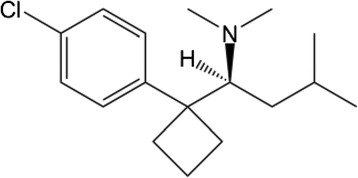
Molecular structure of sibutramine

**Fig. 2:**
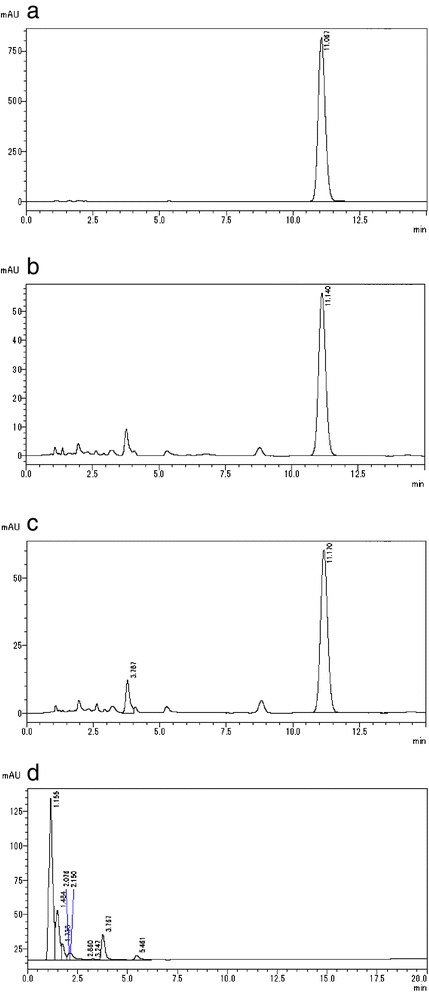
Chromatogram in HPLC analyses under Method 1. **a** Reference standard of sibutramine; **b** item number 1; **c** item number 10; and **d** item number 14

**Fig. 3:**
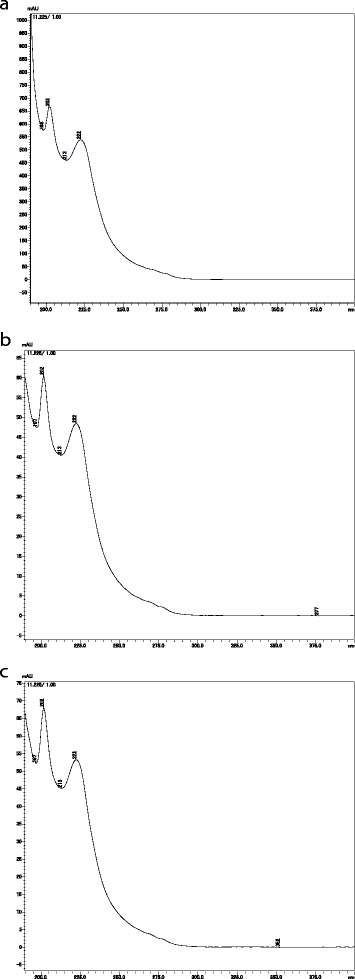
Absorbance spectrum of the peaks that appeared in HPLC analyses under Method 1. **a** Reference standard of sibutramine; **b** the peak that appeared in HPLC analyses of item number 1; and **c** the peak that appeared in HPLC analyses of item number 10

We categorized the 15 items into three types of dosage form: there were eight soft-capsule samples, six hard-capsule samples, and one sugarcoated-tablet sample. One soft-capsule sample could not be analyzed because of an insufficient number of capsules. All soft-capsule samples were filled with a clear, oily liquid. In the chromatogram of this fluid dissolved in the mobile phase for HPLC analyses, we found that the analytic procedure (Method 1) revealed a peak. The retention time of this peak was ≈ 11 min, which is consistent with sibutramine hydrochloride (Figs. 1, 2a, b). In addition to the retention time, the absorbance spectrum of this peak showed close similarity to that of the reference standard of sibutramine hydrochloride (Fig. 3a, b). No other isolated peak was detected. All seven soft-capsule samples, which were not described as containing sibutramine, showed this characteristic chromatogram.

All hard-capsule samples had fawn- or tan-colored granular contents, which contained plant fragments as according to microscopic examination. After dissolving these contents in methanol, HPLC Method 1 revealed a peak at ≈ 11 min (Fig. 2c). The retention time and absorbance spectrum of this peak showed a close similarity to that of the reference standard sibutramine hydrochloride (Figs. 2a, c, 3a, c). All six hard-capsule samples showed this characteristic chromatogram. Other distinct peaks of the crude drug components appeared over 1–2.5 min.

**Fig. 4:**
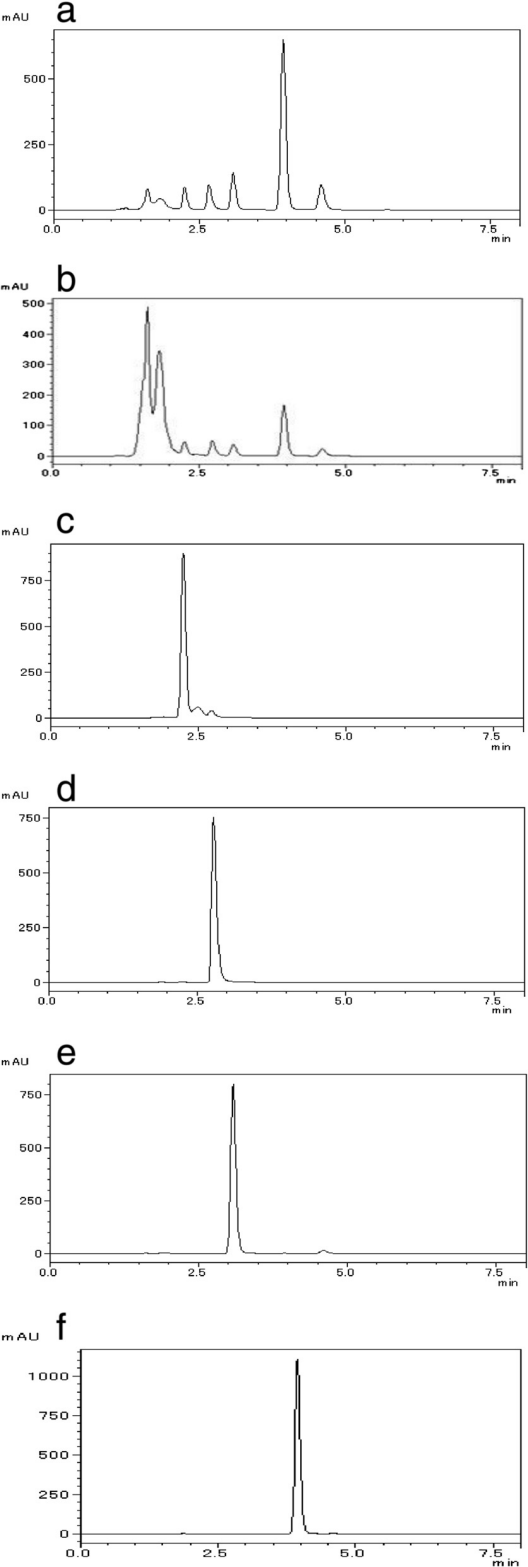
Chromatogram in HPLC analyses under Method 2. Item number 14: an extract of *rhubarb rhizome* and reference standards of anthraquinones in *rhubarb rhizome* were analyzed by HPLC under Method 2. **a**, **b**, **c**, **d**, **e**, and **f** show, respectively, the chromatogram of item number 14, and the extracts of *rhubarb rhizome*, aloe-emodin, rhein, emodin, and chrysophanol

**Fig. 5:**
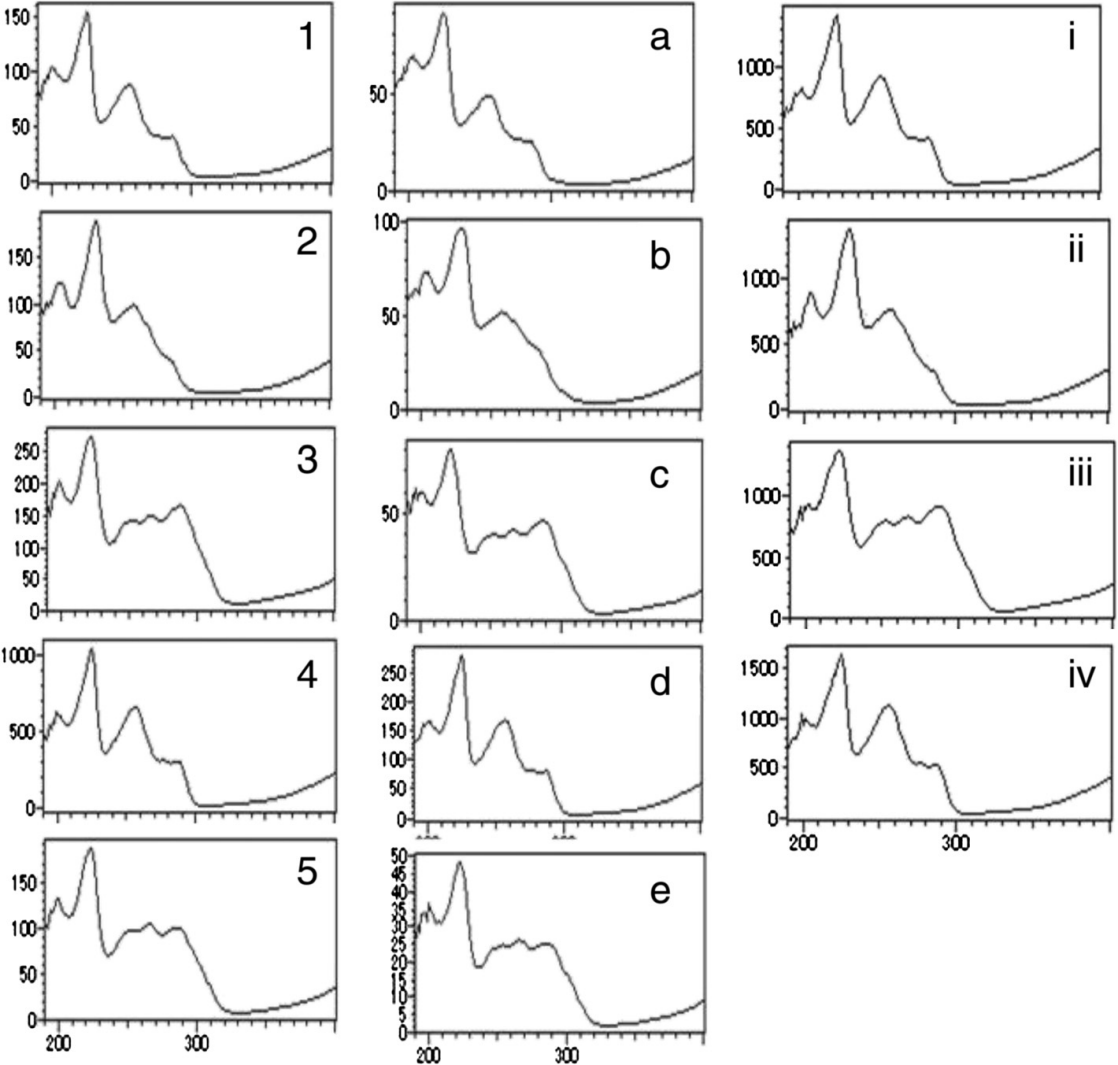
Absorbance spectrum of the peaks that appeared in HPLC analyses under Method 2. The absorbance spectrum of five types of peaks appeared in the chromatogram of item number 14 (1–5) and the extract of *rhubarb rhizome* (**a**–**e**); reference standards of aloe-emodin (i), rhein (ii), emodin (iii), and chrysophanol (iv) are shown

After removing its coating, the one sugarcoated tablet preparation (number 14) was found to have brown contents. In the TLC analyses, sennoside A (the subject of identification of rhubarb rhizome in the *Japanese Pharmacopoeia 16*) was not detected in the extract of this item, whereas the spot derived from sennoside A appeared in the extract of rhubarb rhizome (data not shown). In HPLC analyses under Method 1, some peaks appeared at 1–5 min; no peak was detected at ≈ 11 min in the chromatogram of this extract with methanol (Fig. 2d); no peaks appeared after 5 min when analyses were undertaken with the coating agents removed. The diluent with methanol showed some peaks in the chromatogram under Method 2, and these peaks were consistent with the pattern of extract of rhubarb rhizome (Fig. 4a, b). The retention time and absorbance spectrum of four of the five types of peaks (Fig. 5-1, 5-2, 5-3, 5-4, 5-5, 5a, b, c, d, e) showed a close similarity to those of the reference standards of aloe-emodin (Figs. 4c, 5i), rhein (Figs. 4d, 5-ii), emodin (Figs. 4e, 5-iii), and chrysophanol (Figs. 4f, 5-iv), but one of them was not known (Figs. 4a, b, 5-5, 5e). The constituent of another peak could not be identified.

In HPLC analyses under Method 3, aristolochic acid, fenfluramine, and N-nitroso-fenfluramine were not detected in any item.

For items containing sibutramine, quantitative analyses were carried out using the HPLC-UV method. The mean quantity of sibutramine in each item was 12.37 ± 4.63 mg; the minimum and maximum were ≈ 6 and ≈ 25 mg/capsule, respectively (Table 2). In one item (number 8), the quantity of sibutramine varied among capsules.

**Table 2:** Sibutramine quantities

Product number	Type of dosage form	Quantity (mg/unit)	Authenticity	Reason
1	Soft capsule	11.48 ± 0.61	Falsified (suspected)	Mislabeled
2	Soft capsule	13.43 ± 0.81	Falsified (suspected)	Mislabeled
3	Soft capsule	10.18 ± 1.63	Falsified (suspected)	Mislabeled
4	Soft capsule	10.85 ± 0.25	Falsified (suspected)	Mislabeled
5	Soft capsule	13.13 ± 0.41	Falsified (suspected)	Mislabeled
6	Soft capsule	6.26 ± 2.14	Falsified (suspected)	Mislabeled
7	Soft capsule	10.19 ± 8.52	Falsified (suspected)	Mislabeled
8	Hard capsule	25.85 ± 1.43	Falsified (suspected)	Mislabeled
9	Hard capsule	15.53 ± 1.91	Falsified (suspected)	Mislabeled
10	Hard capsule	10.03 ± 0.97	Falsified (suspected)	Mislabeled
11	Hard capsule	9.67 ± 0.41	Falsified (suspected)	Mislabeled
12	Hard capsule	12.82 ± 1.02	Falsified (suspected)	Mislabeled
13	Hard capsule	11.38 ± 0.17	Falsified (suspected)	Mislabeled
14	Sugarcoated tablet	Not detected	Genuine	The manufacturer did not or provide results of chemical analyses
15	Soft capsule	Not tested because of an insufficient number of capsules	Unknown	

The quantity of sibutramine is represented as the mean ± SD (*n* = 3)

## Discussion

We evaluated the quality of imported anti-obesity medicines comprising crude drugs purchased *via* the Internet. Anti-obesity medicines sold on the Internet include synthetic medicines (e.g., sibutramine hydrochloride, orlistat) as well as crude-drug products and supplements, which are advertised as promoting weight loss. One purchased item containing rhubarb rhizome was identified as a prescription medicine in our authenticity investigation and the others were advertised as dietary supplements that were matched with package information. After HPLC analyses, we determined that the main ingredient of all soft- and hard-capsule samples was the synthetic anti-obesity agent sibutramine (Figs. 2a, b, c, 3), but this information was not described on the package, package insert and/or the website except for one item “代代花胶嚢 (item number 9)”. On the website advertising 代代花胶嚢, we found a description stating that a weight-loss effect owing to sibutramine could be expected (Table 1). Hence, the items confirmed to contain sibutramine could be classified as “falsified products” according to the definition of Attran and colleagues [3]. For the soft-capsule samples, only sibutramine was detected and no crude drugs were found. Among those 13 items, each capsule contained ≈ 12 mg of sibutramine, which was equivalent to or exceeded the recommended daily dose (10 mg) for an anti-obesity medicine. One capsule contained 25 mg of sibutramine.

By contrast, the one sugarcoated tablet preparation did not contain sibutramine hydrochloride, benfluorex, lovastatin, rimonabant, or orlistat (Fig. 2d). The assayed sample of the sugarcoated tablet was found to contain quantities of the major constituents of indicated crude drugs such as the anthraquinones (except for sennoside A) in rhubarb rhizome; the plant fragments in the sample could not be identified (Figs. 4, 5).

These findings indicate that sibutramine was present in most of the herbal supplements that we purchased online and which we assayed. Even supplements that are described as containing only crude drugs should be used carefully because they might contain synthetic adulterants. This is especially true for products purchased from the Internet, for which there is often a lack of compliance to national regulatory standards. Our results indicate that the diet-aid products that we examined may exert an anti-obesity effect as a result of the sibutramine, they contained and not through the crude drugs themselves.

With regard to item number 15 (which could not be evaluated because of an insufficient number of capsules), we detected sibutramine in another item (number 3) that had the same product name, lot number, date of manufacture, and which was sent from the same dispatcher. Thus, sibutramine may have been present in that item.

Sibutramine products are not approved for use in Japan, but they are among the most popular anti-obesity agents sold on the Internet. Sibutramine is a serotonin reuptake inhibitor and noradrenaline reuptake inhibitor, and it induces weight loss through a combination of reduced appetite, feelings of satiety and, possibly, the induction of thermogenesis [23, 24]. However, some adverse cardiovascular events in patients treated with sibutramine have been reported [25]. Following data obtained from the Sibutramine Cardiovascular Outcomes Trial, sibutramine was withdrawn from the market in the USA, Canada, European Union and UK in 2010. The Committee for Medicinal Products for Human Use in the European Medicines Agency recommended that all marketing authorization for medicines containing sibutramine be suspended across the European Union [26–30]. Weight-loss medicines such as herbal supplements disguised as diet-aid products can be acquired readily on the Internet. Some of those products may include harmful substances (e.g., sibutramine), so medicines and supplements purchased on the Internet can damage health.

Medicines may be categorized into two basic types: synthetic medicines and herbal medicines compounded from crude drugs. Often, herbal medicines and supplements are purported to have a lower prevalence of adverse effects than synthetic medicines. However, health problems caused by Chinese herbal medicines containing aristolochic acid have been reported [20, 21, 31, 32]. Since 2002, in Japan, over 700 people (mostly women) have developed health problems after taking unapproved Chinese diet-aid products containing N-nitroso-fenfluramine [22]. In the present study, we found that herbal supplements purchased on the Internet contained sibutramine (a synthetic anti-obesity drug) but not aristolochic acid, fenfluramine, or N-nitroso-fenfluramine. These herbal supplements may be regarded as falsified products because they contained sibutramine, which was not listed as a component.

Our findings indicate clearly that herbal products purchased on the Internet carry the risk of adverse effects. It is necessary to halt the increase in the number of individuals becoming ill after taking medicines purchased on the Internet. Warnings need to be given about the careless use of products disguised as “weight-loss medicines” sold over the Internet. Consumers need to become more fully aware of the risks involved if they purchase such products from the Internet.

## Conclusions

Products confirmed to be supplements and which underwent chemical analyses contained sibutramine, which can exert adverse cardiovascular events. Herbal products that contain harmful substances are distributed worldwide and any individual can obtain them through the Internet, even though the dangers of purchasing medicines and/or supplements online have been documented [33, 34]. These products pose serious safety risks [20–22, 35]. These fraudulent products should not be sold on the Internet but, for the moment, consumers should protect themselves by avoiding inappropriate use of pharmaceutical agents. To expedite access to genuine products for consumer safety, improved regulation of illegitimate medicines and supplements, increased inspection and guidance to comply with existing rules, and international cooperation by public-health agencies, drug regulatory bodies and law enforcement agencies could help ensure secure pharmaceutical distribution. Governments and individual consumers need to be more vigilant regarding the possible health risks associated with use of these bogus herbal preparations.

## Abbreviations

(HPLC): High-performance liquid chromatography
(PDA): Photodiode array detector
(TLC): Thin-layer chromatography
(UV): Ultra violet
(WHO): World Health Organization
